# Everolimus Through Plasmatic Concentrations in Cancer Patients: Prospective Longitudinal Observational Multicentric Study (DIANA-1 Project)

**DOI:** 10.3390/jcm14010145

**Published:** 2024-12-30

**Authors:** Eduard Fort-Casamartina, Sonia Pernas, Sara Otero, Paula Mate, Núria Gonzalo, Sonia Narváez, Raúl Rigo-Bonnin, Ariadna Padró-Miquel, Àlex Teulé, Xavier Garcia del Muro, Inma Peiró, Lorena Arribas, Anna Esteve, Andrea Gonzalez, Montse Rey, Ana Clopés, Sandra Fontanals, Carme Muñoz

**Affiliations:** 1 Pharmacy Department, Institut Català Oncologia (ICO), Institut d’Investigació Biomèdica de Bellvitge (IDIBELL), L’Hospitalet Llobregat, 08908 Barcelona, Spain; saraotero@iconcologia.net (S.O.); pmate@iconcologia.net (P.M.); ngonzalo_ext@iconcologia.net (N.G.); snarvaez@iconcologia.net (S.N.); morey@iconcologia.net (M.R.); sfontanals@iconcologia.net (S.F.); cms@iconcologia.net (C.M.); 2 Medical Oncology Department, Institut Català Oncologia (ICO), Institut d’Investigació Biomèdica de Bellvitge (IDIBELL), L’Hospitalet Llobregat, 08908 Barcelona, Spain; spernas@iconcologia.net (S.P.); ateule@iconcologia.net (À.T.); garciadelmuro@iconcologia.net (X.G.d.M.); 3 Laboratory of Molecular Genetics, Laboratori Clínic Territorial Metropolitana Sud, Hospital Universitari de Bellvitge, Institut d’Investigació Biomèdica de Bellvitge, Institut d’Investigació Biomèdica de Bellvitge (IDIBELL), L’ Hospitalet Llobregat, 08908 Barcelona, Spain; rigo@clinic.cat (R.R.-B.); apadro@bellvitgehospital.cat (A.P.-M.); 4 Clinical Nutrition Unit, Institut Català Oncologia (ICO), Institut d’Investigació Biomèdica de Bellvitge (IDIBELL), L’Hospitalet de Llobregat, University of Barcelona, 08908 Barcelona, Spain; vpeiro@iconcologia.net (I.P.); larribas@iconcologia.net (L.A.); 5 Medical Oncology Department, Institut Català Oncologia (ICO), Badalona Applied Research Group in Oncology (B-ARGO), Germans Trias I Pujol Research Institute (IGTP), 08916 Badalona, Spain; aesteve@iconcologia.net (A.E.); andreagonzalez@iconcologia.net (A.G.); 6 Research Management Unit (UGR), Institut Català Oncologia (ICO), 08916 Badalona, Spain; 7 CatSalut Medicine Area Director, 08028 Barcelona, Spain; aclopes@catsalut.cat

**Keywords:** everolimus, therapeutic drug monitoring, pharmacokinetic variability, optimal dosage, cancer

## Abstract

**Background:** Everolimus, an oral inhibitor of the mammalian target of rapamycin (mTOR), is actually used to prevent organ transplant rejection and treat metastatic breast, renal, and neuroendocrine cancers. Despite significant pharmacokinetic variability among patients, routine therapeutic drug monitoring (TDM) is not commonly used in oncology. **Methods:** The aim of this multicenter, prospective observational cohort study is to assess the prevalence of everolimus minimum concentration at a steady state (Cminss) falling outside the therapeutic range (10–26.3 ng/mL) during a routine TDM programme. Sixty patients with metastatic breast, neuroendocrine, or renal cancers, either starting or continuing everolimus treatment according to hospital protocols, are to be included between 1st of January 2024 and 31st of December 2025 (patients undergoing clinical trials are excluded). We hypothesize that 30–50% of our patients and their blood samples will not achieve the target optimal plasma concentrations. Blood samples are collected every 4–6 weeks to monitor drug levels. The secondary goal is to explore correlation between out-of-range everolimus levels and factors such as demographic and anthropometric data, treatment specifics, lab results, genetic polymorphisms, and the presence of toxicity. **Conclusions:** This study could offer valuable insights into optimizing dosing strategies and may contribute to future research on personalizing everolimus and other anticancer treatments. This personalized approach seeks to tailor therapy not only to the tumour’s molecular profile but also to the individual characteristics of each patient, improving both drug selection and dosing precision.

## 1. Background

Over the past two decades, several oral anticancer drugs have significantly improved median progression-free survival and overall survival across various tumour types. In contrast to chemotherapy or immunotherapy, most of these treatments are still being used at fixed doses using a “oneIt0dose-fits-all” approach, where drug selection is primarily based on the tumour’s molecular characteristics. Consequently, a substantial proportion of patients may be overtreated (>15%) or undertreated (>30%) at the labelled doses [1].

Everolimus, an oral inhibitor of mammalian target of rapamycin (mTOR), is commonly used to prevent solid organ transplant rejection and treat metastatic breast, renal, and neuroendocrine cancers. Due to its narrow therapeutic index, therapeutic drug monitoring (TDM) is routinely used in transplant patients [2] to ensure optimal dosing. The optimal steady-state minimum concentration (Cminss) should generally be targeted within the range of 3–8 ng/mL when used in conjunction with other immunosuppressive agents, such as calcineurin inhibitors or glucocorticoids. For regimens that do not include calcineurin inhibitors, the target Cminss should be between 6 and 10 ng/mL.

In oncology, however, TDM has not yet been integrated into routine clinical practice [3], despite the higher fixed daily dose of 5 or 10 mg. Dose reductions, interruptions, and discontinuations are frequently needed due to toxicity (e.g., stomatitis, diarrhea, noninfectious pneumonitis, asthenia, laboratory abnormalities, and infections). A meta-analysis by Ravaud et al. [4] found that the mean everolimus Cminss was 15.65 ng/mL (90%CI 14.79–16.55 ng/mL). A two-fold increase in Cminss was associated with a better response and more significant tumour size reduction. Thus, a Cminss ≥10 ng/mL could serve as a target for optimal therapeutic response, while levels >26.3 ng/mL are linked to a four-fold increase in the risk of toxicity.

In a multicenter study conducted by Groenland [1] involving 600 patients treated with 24 different oral antineoplastic drugs, 1647 out of 2536 blood samples (64.9%) showed steady-state minimum concentrations (Cminss) below the predefined target levels. Pharmacokinetic interventions were successful in 113 of 152 patients

Several factors may contribute to the inter-individual pharmacokinetic variability of everolimus exposure, including treatment adherence, sex, age, drug–drug and food–drug interactions, physiological conditions (dysphagia, malabsorption, altered stomach pH), genetic polymorphisms affecting metabolism or efflux pump proteins, and body composition.

The DIANA-1 Project is a multicentric study aimed at analyzing and identifying patients with metastatic breast, renal, or neuroendocrine cancers who are treated with everolimus but exhibit mean Cminss outside of the therapeutic range. This study will analyze several factors contributing to this variability. The insights gained from this research could pave the way for future efforts to individualize everolimus treatment based on patient-specific characteristics, ensuring that patients receive the appropriate drugs and the correct dosages.

## 2. Material and Methods

### 2.1. Study Objective

The main objective of the present study is to evaluate the prevalence of patients and samples with Cmin values out of therapeutic range in metastatic breast, neuroendocrine, and renal cancer patients treated with everolimus. 

The secondary objective is to identify associations between the everolimus plasmatic concentration out of range according to demographic variables, anthropometry, treatment characteristics, laboratory parameters, genetic polymorphisms, and the presence of toxicity.

### 2.2. Hypothesis

The doses of everolimus typically used are as follows: 5 or 10 mg once daily (QD) in breast, renal, and neuroendocrine metastatic cancer patients. However, due to high inter and intraindividual variability, 30–50% of our patients and blood samples could not reach mean optimal plasmatic concentrations. 

### 2.3. Study Design and Settings

This is a prospective longitudinal observational multicentric study including breast, renal, and neuroendocrine metastatic cancer patients who start or are undergo everolimus treatment. Given the descriptive observational prospective cohort, we plan to recruit a sample size of 60 patients. We will be prospectively including subsequent patients fulfilling eligibility criteria between 1st January 2024 and 31st of December 2025.

### 2.4. Ethics

The Ethics Committee of the Bellvitge University Hospital approved the DIANA-1 study protocol (Reference number EOM033/23). The study is conducted following the Good Clinical Practice guidelines and the provisions of the Declaration of Helsinki. All patients are provided with information about the study, and written informed consent is obtained prior to their inclusion.

### 2.5. Inclusion Criteria

The study population consists of adult patients with metastatic breast, lung, or neuroendocrine cancer who start or continue everolimus treatment according to hospital protocols outlined by Eastern Cooperative Oncology Group (ECOG) 0-2. Everolimus must be administered in the morning in fast conditions (a minimum one hour before breakfast). Patients are treated at Institut Català of Oncologia (Hospitalet, Badalona, Girona). All blood samples must be taken within hospitals involved, and pharmacogenetic and pharmacokinetics determinations are centralized to “Laboratori Clínic Metropolitana Sud” of Bellvitge Hospital. The study population includes patients aged 18 years or older who are registered with Eastern Cooperative. The study’s fulfilment will be reached in less than two years.

### 2.6. Exclusion Criteria

Exclusion criteria include swallowing difficulties that prevent patients from taking everolimus tablets, which may be due to dysphagia or another physiological condition; cases where blood samples cannot not be analyzed in recruitment centres; and patients whose everolimus use is under the clinical trial protocol.

## 3. Study Procedures

### 3.1. Everolimus Treatment

Everolimus treatment is prescribed by a medical oncologist for a (28–42)-day cycle and is validated by an oncological hospital pharmacist, reviewing treatment indications, the line of therapy, metastasis localization, the everolimus dose, treatment reductions and interruptions, adherence, and relative dose intensity.

Pharmaceutical care is provided at the inclusion interview and follow-up visits every 4–6 weeks (Table 1). Written and oral information about everolimus treatment is given, and all patients are instructed to take it in fasting conditions in the morning, one hour before breakfast, but on the day of the analysis.

### 3.2. Adherence and Relative Dose Intensity

Adherence and relative dose intensity (RDI) are calculated in each everolimus blood determination via pharmacist interview. The WHO definition of adherence is as follows: “the extent to which a person’s behaviour—taking medication, following a diet, and/or executing lifestyle changes, corresponds with agreed recommendations from a health care provider” [5]. We register each everolimus delivery using our electronic software and adherence is calculated in each cycle of prescription by dividing the amount of medication actually taken by the patient by the amount they should have taken in a period of time. Good adherence to everolimus is defined as between 90 and 110%. Adherence follow-up could be one of the limitations of the study: this involves the assumption that once the medication is dispensed to the patient, they take it correctly at home and that the information provided by them is reliable.

$$\%\;\mathrm{adherence}=\frac{\mathrm{amount}\;\mathrm{of}\;\mathrm{medication}\;\mathrm{taken}}{\mathrm{amount}\;\mathrm{of}\;\mathrm{medication}\;\mathrm{should}\;\mathrm{have}\;\mathrm{taken}}\times 100$$

The global relative dose intensity (RDI), as well as that of each cycle, is calculated as the received dose expressed as a percentage of the standard dose (10 mg once daily) throughout the treatment and after each everolimus dose [6].

$$\mathrm{RDI}=\frac{\mathrm{Total}\;\mathrm{milligrams}\;\mathrm{really}\;\mathrm{administered}}{\mathrm{Total}\;\mathrm{days}\;\mathrm{treated}\;\times 10\;\mathrm{mg}}\times 100$$

### 3.3. Pharmacological Interaction

In each pharmaceutical follow-up visit, pharmacists in the Out-Patient Pharmacy Unit check and register the generic names of all prescribed drugs. The Drugs^®^ data base is used to detect potential drug–drug interactions (DDIs) with everolimus. DDIs [7] are classified as major or moderate interactions, and several CYP3A4 inhibitors, CYP3A4 inductors, P-glycoprotein inhibitors, angiotensin-converting enzyme inhibitors, and antiacids drugs (proton pump inhibitors, histamine receptor antagonists, and others) are registered.

### 3.4. Everolimus Therapeutic Drug Monitoring

Pharmacokinetic everolimus monitoring is performed before starting each everolimus cycle. Patients are considered at a steady state after four to five times the everolimus half-life time (t1/2 = 30 h), and they must have been treated for at least 14 consecutive days before determination. Cminss blood samples are obtained 22–26 h after the last everolimus dose. The optimal therapeutic range for everolimus concentrations is between 10ng/mL and 26.3ng/mL. All Cminss values are blinded until the end of the study, and a mean value is calculated for each patient.

An ultra-high-performance liquid chromatography device, coupled to a mass spectrometry device, is used for (UHPLC-MS/MS) for the determination of everolimus concentrations. It was previously developed and validated by the Rigo-Bonnin Group [8]. The everolimus blood concentration is detected by ESI mass spectrometry in a positive ion multiple-reaction monitoring mode using a mass-to-charge transition of 975.5→908.3/891.6.

### 3.5. Genetic Variants

An automated DNA purification system is used to extract DNA from peripherial blood (Maxwell^®^ RSC Instruments, Promega, Madison, WI, USA). The DNA concentration is obtained by Quantus Fluorometer using QuantiFluor^®^ ONE dsDNA System (Promega Madison, WI, USA) and stored at −80 °C.

The following five single-nucleotide variants (SNVs) located in genes involved in everolimus metabolism (*CYP3A4* and *CYP3A5* genes), everolimus transport (*ABCB1* gene), or the PI3K/AKT/mTOR pathway (*PIK3R1* and *RAPTOR* genes) are selected for genotyping according to allele frequency and scientific evidence (Table 2) [9].

Genotyping is performed using the TaqMan SNV Genotyping Assay (assay IDs: C___7586657_20, C___3164019_10, C__59013445_10, C__26201809_30) and Custom TaqMan Assays from Thermo Fisher Scientific (Waltham, MA, USA). The assays are set up in 96-well plates, with both positive and negative controls included. Real-time PCR is conducted on the QuantStudio 3 qPCR System (Thermo Fisher Scientific, Waltham, MA, USA) following standard procedures. Specifically, 1 µL of Assay Mix is combined with 10 µL of Supermix SsoAdvanced (BioRad^®^, Hercules, CA, USA), 2 µL of genomic DNA (30 ng/µL), and purified water to reach a final volume of 20 µL. The thermal cycling protocol involves heating the mixture to 50 °C for 2 min, followed by heating at 95 °C for 10 min. This is succeeded by 40 cycles of denaturation at 95 °C for 15 s, and annealing/extension at 60 °C for 60 s.

### 3.6. Analytical Determination

The hematological and chemistry blood profiles are determined in each everolimus cycle. This includes glucose, insulin, renal clearance as assessed by the Cockcroft–Gault equation, albumin, prealbumin, sodium, potassium, calcium, phosphate, magnesium, hepatic functions, total cholesterol, HDL cholesterol, LDL cholesterol, triglyceride, lactate dehydrogenase, C-reactive protein, hemoglobin, glycated hemoglobin, absolute neutrophils, platelets, leucocytes, and the lymphocyte count.

### 3.7. Body Composition and Anthropometry

**Figure 1:**
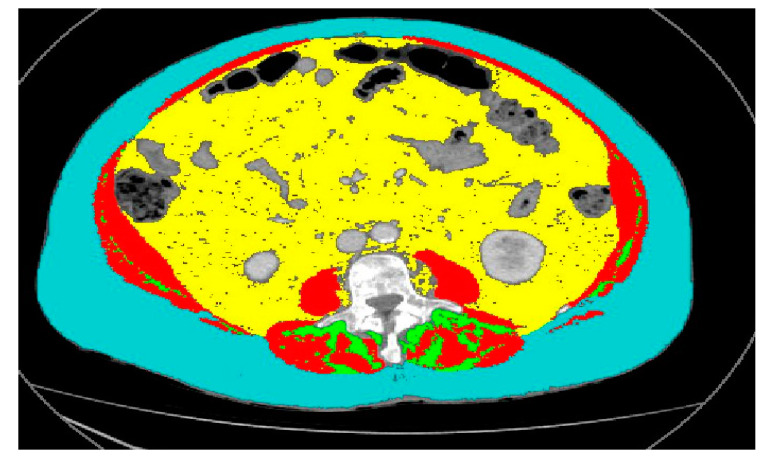
Example of PET/CT body composition analysis. Red: Muscle Mass; Blue: Subcutaneous Adipose Tissue; Yellow: Visceral Adipose Tissue; Green: Intramuscular Adipose Tisue.

Body composition at baseline and during follow-up (every 3–6 months) is analyzed using positron emission tomography with a computed tomography scanner (PET/CT) technique. Based on previous reports about this level, the third lumbar (L3) vertebra is chosen for the axial cross-section CT (Figure 1) component of the whole-body PET/CT scans as the reference point based on previous reports, with this level used to calculate the skeletal muscle index (SMI) [10,11]. Muscle mass is quantified within a Hounsfield unit (HU) range from −29 to +150HU using SliceOmatic© software (v5.0 Rev 8, Tomovision, Montreal, Quebec, Canada). Cross-sectional adipose tissue areas are determined using the tissue-specific HU range defined at this level [11,12]. Muscle mass and total fatty tissue (including visceral and subcutaneous) are quantified. The muscle cross-sectional area is then normalized for height and reported as SMI (cm^2^/m^2^). The estimated kilogrammes of SMM and FM are calculated from regression equations reported by Shen et al. [11]. At baseline and follow-ups, the current body weight, 6-month previous weight, the body mass index, and the body surface area (Dubois and Dubois equations) are also recorded [10,11,12].

### 3.8. Samples Preparation and Storage

Blood samples are collected alongside routine safety laboratory assessments performed during standard follow-up visits, resulting in minimal additional burden for patients. Extra 10 mL whole-blood samples are obtained for pharmacogenetic and pharmacokinetic determinations. Samples are stored between 2 and 8 °C and aliquoted during the following 48 h. The 6–7 aliquots of each sample are stored at −80 °C for up to 90 days until their analysis [13].

### 3.9. Statistical Analysis 

A descriptive analysis will be performed. Frequencies and percentages are used to express qualitative variables and quantitative variables using the mean or median values and their respective 95% confidence intervals or interquartile ranges, respectively. The prevalence of patients with everolimus plasma concentrations falling outside the therapeutic range will be computed as the number of patients with a mean of Cmin values not achieving the optimal therapeutic range during follow-up out of the total number of patients included in the study. The prevalence of blood samples falling outside of the therapeutic range will be computed as the number of blood samples with a Cmin value not achieving the optimal therapeutic range out of the total number of samples. The comparison between groups of patients achieving or not achieving the optimal therapeutic range will be performed using the chi-square test for the qualitative variables and Student’s *t*-test, or the Kruskal–Wallis non-parametric test, for the quantitative ones. Logistic regression models will be used to identify risk factors associated with the probability of failing outside the therapeutic range. Odds ratios and their 95% confidence intervals will be reported. The statistical analysis will be conducted using R software v. 4.1.2.

### 3.10. Planned Study Period

The recruitment period for this study is expected to take 12–24 months. The first patient was included in January 2024.

## 4. Data Management

Study data will be collected and managed using REDCap electronic data capture tools from Institut Català d’Oncologia. REDCap (Research Electronic Data Capture) is a secure, web-based software platform designed to support data capture for research studies [14,15], providing (1) an intuitive interface for validated data capture; (2) audit trails for tracking data manipulation and export procedures; (3) automated export procedures for seamless data downloads to common statistical packages; and (4) procedures for data integration and to ensure interoperability with external sources.

## 5. Discussion

Everolimus is an oral inhibitor of mTOR that prevents activation after binding with high affinity to the FK506-binding protein-12 (FKBP-12) complex.

In metastatic breast, renal, or neuroendocrine tumours, the initial doses of everolimus usually are 5 or 10 mg QD.

Dose reductions, dose interruptions, or discontinued treatment are needed in 10–35% of patients due to adverse events (stomatitis, diarrhea, noninfectious pneumonitis, asthenia, biological abnormalities, and infections) [16,17,18].

Everolimus is rapidly absorbed in cancer patients, and peak blood concentrations (Cmax) are reached 1–2 h after his daily administration of 5–10 mg daily with a high distribution volume and an elimination half-life of 30 h (in hepatic impairment increases until 77 h) [19]. The cytochrome P450 (CYP) complexes CYP3A4, CYP3A5 and CYP2C8 are the primary enzymes involved in everolimus metabolism and they are also substrates for the efflux pump P-glycoprotein.

In a meta-analysis by Ravaud et al. [4], the mean everolimus Cminss was 15.65 ng/mL (90%CI 14.79–16.55 ng/mL). A better response and a significant reduction in tumour size were observed with a two-fold increase in Cminss. In conclusion, Cminss ≥ 10 ng/mL can be used as the target value to achieve an optimal response [4], while Cminss > 26.3 ng/mL is associated with a four-fold increased risk of toxicity [20].

The rates of patients with neuroendocrine, lung, and renal cancer with non-optimal Cminss were 55%, 44.8%, and 37.1%, respectively, and higher mPFS were oberved in patients with Cminss between 10 and 30 ng/mL [4].

In solid transplant patients, everolimus TDM is routinely used to achieve optimal Cmins values, whereas, in cancer patients, its use is not established, although higher doses are used and several factors can impact the everolimus pharmacokinetic profile:

- Age: In a phase III study analysis [21], significant age-related differences were detected in cancer renal patients receiving adjuvant everolimus. Cminss everolimus values were 14.4 (1.7, 70.5), 18.4 (0.5, 60.7), and 20.8 (0.3, 75.6) ng/mL < 52 years, 52–61 years, and ≥ 62 years, respectively. A correlation index of 0.4049 was found in renal cancer patients [22].
- Sex: Significant sex-related differences were also observed in everolimus adjuvant treatment. Everolimus Cminss was significantly higher in menn than women (19.4 versus 15.4 ng/mL, *p* = 0.01) [21].
- Administration conditions: After a single 10 mg dose of everolimus in 24 patients, the maximum concentration (Cmax) and the Area Under the Curve (AUC) were reduced by 42% and 22%, respectively, when compared to fasting conditions after low-fat meals. These reductions increased to 54% and 33% following a high-fat meal [23].
- Adherence treatment: Several studies have been conducted on treatment adherence in chronic myeloid leukemia, hormone therapy (breast or prostate cancers), and capecitabine (digestive or breast cancers in those over 65 years). Adherence rates are highly variable, ranging from 46% to 100% [24,25,26], depending on the patients, the type of oral anticancer therapy used, the follow-up period, the definition of adherence, and the method of measurement.
- Relative dose intensity: Total dose administered during treatment divided by the initial standard dose intensity specified in the protocol. Due to treatment-related toxicities or poor adherence, relative dose intensity could be lower than 100% [27,28].
- Body composition/anthropometry: An altered body composition may cause modifications in drug pharmacokinetic profile. In a metanalysis by Gerard et al. [29], a higher risk of toxicities grade III-IV and dose reductions were observed in sarcopenic cancer patients: HR 13.5 (IC95% 1.08–169.3) and HR 2.95 (IC95% 1.23–7.1). Statistically higher Vandetanib serum concentrations and probabilities of toxicities were observed in sarcopenic medullary thyroid carcinomas [30]. Renal cancer patients with skeletal muscle index values in the highest tercile had significantly better overall survival: 21.9 vs. 10 months (*p* = 0.0025) [31]. In neuroendocrine cancer patients [32], shorter progression-free survival was observed with low muscle and fatty index and a body mass index ≤ 18.49 kg/m^2^.
- Hypoalbuminemia: Plasma everolimus predominantly binds to albumin [7]. Factors affecting protein binding, such malnutrition, can increase drug toxicity because the greater the free fraction in plasma, the more pronounced the effect of the drugs.
- Drug–drug interactions: The pharmacokinetic characteristics of oral anticancer drugs could be affected by drug–drug interactions, affecting primarily metabolism and distribution. Polymedicated patients, defined as patients who are treated with more than 5 drugs, are more likely to experience pharmacological interactions. The significant/moderate induction or inhibition of cytochrome P450 (CYP450), P-gp transport, or both is important over the course of everolimus pharmacokinetic treatment. Interactions with CYP3A4 inhibitors, like verapamil, clarithromycin, erythromycin, voriconazole and CYP34 inducers, like fenofibrate, are reported [33,34,35,36,37,38].
- Genetic variants: In oncology, it seems that the *CYP3A5* genotype has no effect on everolimus Cminss, but a statistically higher Cminss value is observed in CYP3A4*22 carriersvs wild-type patients (*p* = 0.019.). On the other hand, polymorphisms in genes of the mTOR pathway may be responsible for variations in EVR efficacy or associated with the occurrence of adverse events [9,39].

## 6. Conclusions

The proposed study aims to identify patients who do not achieve optimal Cminss everolimus treatment in routine clinical daily practice when therapeutic drug monitoring (TDM) is not used. This study will explore their correlation with everolimus plasm exposure by incorporating various individual factors into a comprehensive analysis. The findings may highlight TDM as a valuable tool for moving towards the individualization of treatment with targeted oral therapies, ensuring more precise and effective dosing that is tailored to each patient’s needs.

## Figures and Tables

**Table 1:** Study variables.

Variable	Inclusion (Visit 0)	Follow-Up	Follow-Up
		(Visit 1)	(Visit > 1)
**Demographic**			
- Sex	X		
- Date of birth	X		
- Date of inclusion	X		
- Civil status	X		
- Academic training	X		
- Smoking history	X		
- Comorbidities	X		
- Weight	X	X	X
- Height	X		
- Body composition	X		X
**Oncological History**			
- Cancer diagnostic	X		
- Date of metastatic diagnostic	X		
- Type of metastases	X		
- Line of treatment	X		
- Start or ongoing everolimus treatment	X		
- Start everolimus date	X		
- Date of first metastatic line treatment	X		
**Pharmacogenetic analysis**			
- rs1045642 ABCB1		X	
- rs10515074 PIK3R1		X	
- rs9906827 de RAPTOR		X	
- rs35599367 CYP3A4		X	
- rs776746 CYP3A5		X	
**Everolimus pharmacokinetic**			
- Date/hour of last everolimus administration		X	X
- Fasting conditions		X	X
- Date/hour blood sample		X	X
- Cminss determination		X	X
**Everolimus treatment**			
- Daily dose		X	X
- Adherence		X	X
- Dose intensity		X	X
- Dose reduction		X	X
- mg everolimus/weight		X	X
- mg everolimus/BSA		X	X
- Current weight		X	X
- Previous 6-month weight		X	X
**Chronic treatment**			
- Number of drugs		X	X
- CYP3A4 interactions		X	X
- P-gP interactions		X	X
- Antiacid drugs		X	X
- Number of total interactions		X	X
**Laboratory parameters**			
- Biochemistry		X	X
- Haematologicy		X	X
- Nutritional		X	X
- Lipidic Profile		X	X
- Pro-inflammation		X	X
**Toxicity and grade CTCAE 5.0**			
- Hypertension		X	X
- Hyperglycemia		X	X
- Mucositis		X	X
- Diarrhea		X	X
- Pneumonitis		X	X
- Neutropenia		X	X
- Anemia		X	X
- Thrombocytopenia		X	X
- Edema		X	X
- Anorexia		X	X
- Asthenia		X	X
- Cutaneous		X	X

**Table 2:** SNVs included in the study.

Gene	Gene Category	SNV	Variant Type	Genetic Polymorphisms
				Wild-type: C/C
*ABCB1*	Transport	rs1045642 C>T	Synonymous	Heterozygous: T/C
				Homozygous: T/T
				Wild-type: C/C
*CYP3A4*	Metabolism	rs35599367 C>T (*1/*22)	Intronic	Heterozygous: C/T
				Homozygous: T/T
				Wild-type: G/G
*CYP3A5*	Metabolism	rs776746 G>A (*3/*1)	Intronic	Heterozygous: G/A
				Homozygous: A/A
				Wild-type: A/A
*PIK3R1*	mTOR pathway	rs10515074 A>G	Intronic	Heterozygous: A/G
				Homozygous: G/G
				Wild-type: C/C
*RAPTOR*	mTOR pathway	rs9906827	Intronic	Heterozygous: C/T
				Homozygous: T/T

## Data Availability

No new data were created or analyzed in this study. Data sharing is not applicable to this article.
